# Genetic Background Negates Improvements in Rice Flour Characteristics and Food Processing Properties Caused by a Mutant Allele of the *PDIL1-1* Seed Storage Protein Gene

**DOI:** 10.1186/s12284-022-00560-w

**Published:** 2022-03-05

**Authors:** Kiyosumi Hori, Tomoya Okunishi, Kenji Nakamura, Ken Iijima, Masahiro Hagimoto, Katsuyuki Hayakawa, Koka Shu, Takashi Ikka, Hiroto Yamashita, Masanori Yamasaki, Yoshinobu Takeuchi, Shota Koyama, Yoshimasa Tsujii, Toshiaki Kayano, Takuro Ishii, Toshihiro Kumamaru, Yasushi Kawagoe, Toshio Yamamoto

**Affiliations:** 1 National Agricultural and Food Research Organization (NARO), Tsukuba, 305-8518 Japan; 2 National Institute of Agrobiological Sciences, Tsukuba, 305-8602 Japan; 3 Cereal Science Research Center of Tsukuba, Nisshin Flour Milling Inc, Tsukuba, 300-2611 Japan; 4 Present Address: Faculty of Agriculture, Shizuoka University, Shizuoka, 422-8529 Japan; 5 Food Resources Education and Research Center, Kobe University, Kasai, 675-2103 Japan; 6 Department of Agricultural Chemistry, Tokyo University of Agriculture, Tokyo, 156-8502 Japan; 7 Faculty of Agriculture, Kyushu University, Fukuoka, 819-0395 Japan; 8 Present Address: Institute of Plant Science and Resources, Okayama University, Kurashiki, 710-0046 Japan

**Keywords:** Rice (*Oryza sativa* L.), Seed storage protein mutation, Protein disulfide isomerase, Rice flour characteristics, Food processing suitability, Gene expression, Grain component, Agronomic trait, Genetic background

## Abstract

Phenotypic differences among breeding lines that introduce the same superior gene allele can be a barrier to effective development of cultivars with desirable traits in some crop species. For example, a deficient mutation of the *Protein Disulfide Isomerase Like 1–1* (*PDIL1-1*) gene can cause accumulation of glutelin seed storage protein precursors in rice endosperm, and improves rice flour characteristics and food processing properties. However, the gene must be expressed to be useful. A deficient mutant allele of *PDIL1-1* was introduced into two rice cultivars with different genetic backgrounds (Koshihikari and Oonari). The grain components, agronomic traits, and rice flour and food processing properties of the resulting lines were evaluated. The two breeding lines had similar seed storage protein accumulation, amylose content, and low-molecular-weight metabolites. However, only the Koshihikari breeding line had high flour quality and was highly suitable for rice bread, noodles, and sponge cake, evidence of the formation of high-molecular-weight protein complexes in the endosperm. Transcriptome analysis revealed that mRNA levels of fourteen *PDI*, *Ero1*, and *BiP* genes were increased in the Koshihikari breeding line, whereas this change was not observed in the Oonari breeding line. We elucidated part of the molecular basis of the phenotypic differences between two breeding lines possessing the same mutant allele in different genetic backgrounds. The results suggest that certain genetic backgrounds can negate the beneficial effect of the *PDIL1-1* mutant allele. Better understanding of the molecular basis for such interactions may accelerate future breeding of novel rice cultivars to meet the strong demand for gluten-free foods.

**Supplementary Information:**

The online version contains supplementary material available at 10.1186/s12284-022-00560-w.

## Background

Rice (*Oryza sativa* L.) is a staple food for more than half of the world’s population. Consumer preference causes strong demand for high-quality grain in rice cultivars, although increasing crop yield has long been an important requirement in many rice breeding programs (Champagne et al. 1999; Fitzgerald et al. 2009; Hori and Yano 2013; Custodio et al. 2019). Rice is mainly cooked and eaten as polished (white) rice, but it is also made into flour for noodles, cakes, dumplings, and gluten-free bread. Global improvements in living standards and greater demand for gluten-free foods are increasing the use of rice flours for breads, noodles, and cakes throughout the world (Ashida et al. 2009; Yano 2012; Ashida 2014; Kwak et al. 2017; Fradinho et al. 2019; Paz et al. 2020). However, few of the rice cultivars in current use are suitable for making these products (Sasahara et al. 2013; Ohta et al. 2015; Kwak et al. 2017). Therefore, new rice cultivars with both good processing attributes and high eating quality for various food applications need to be developed to meet consumer preferences, including gluten-free foods.

Many genes affect grain components, and cooking and eating quality (Fiaz et al. 2019; Guo et al. 2020). Recent progress in the genetic analysis of grain quality traits has identified many quantitative trait loci (QTLs) and their underlying genes. Several genes involved in the control of grain quality have been identified so far, but the regulatory mechanisms that contribute to the grain quality traits remain unclear (Zhang et al. 2021; Liu et al. 2022). Genetic control of rice grain quality has been successfully elucidated by gene identification through map-based cloning and the development of mutant lines.

The main components of polished rice grain (endosperm) are starch (up to 95% of dry weight), protein (5–7%), lipids (0.5–1%), as well as minerals, vitamins, and other micronutrients (less than 1%). All of these components affect the cooking characteristics and eating quality (Champagne et al. 1999; Fitzgerald et al. 2009). As the main component of the grain, starch largely controls the cooking characteristics and eating quality, but the seed storage proteins have the second most important effect on these traits (Martin and Fitzgerald 2002; Hori 2018; Hori et al. 2021b). Wheat flour contains the seed storage protein glutenin, which is necessary for the swelling and rising of doughs in breads, noodles, and cakes. Glutenin is converted to high-molecular-weight glutens with three-dimensional reticulate protein structures by inter-molecular disulfide bond formation with other seed storage proteins (gliadins) during food processing (Hu et al. 2021). However, seed storage proteins of rice grain do not form the same three-dimensional reticulate structures as wheat glutenin. This is a serious problem for the development of rice cultivars for flour and gluten-free food production. If the grain proteins in a rice cultivar formed inter-molecular disulfide bonds as effectively as the glutenin and gliadin do in wheat, such a cultivar would be a novel and useful ingredient for making breads, noodles, and cakes.

Rice grain has three classes of seed storage proteins: acid-soluble glutelin, alcohol-soluble prolamin, and salt-soluble globulin (Padhye and Salunkhe 1979; Juliano and Houston 1985). The glutelin in rice is homologous to glutenin in wheat, barley, and rye and to the 11S globulins in soybean and pea (Zhao et al. 1983; Takaiwa et al. 1987). Prolamin is typically found in cereal crops and is homologous to gliadin in wheat (Ogawa et al. 1987; Muench 1999). Globulin is accumulated in some cereal species including oats and rice (Padhye and Salunkhe 1979). Glutelin is initially synthesized on the endoplasmic reticulum (ER) as a 57-kDa precursor that is exported to the protein storage vacuole to form protein body II (PBII); this is converted to acid- and alkali-soluble subunits and post-translationally processed into two subunits linked by intra-molecular disulfide bonds (Yamagata et al. 1982).

Rice has at least 12 genes encoding protein disulfide isomerases (PDI), similar to Arabidopsis and maize (Houston et al. 2005). The *endosperm storage protein 2* (*esp2*) gene was identified in a seed storage protein mutant line of rice that lacks the protein disulfide isomerase-like 1–1 (PDIL1-1) protein (Takemoto et al. 2002). The molecular function of PDIL1-1 is to create the intra-molecular disulfide bonds between two glutelin subunits to facilitate folding of the mature protein into its functional conformation and to enable ER export. Immunoblot analyses to investigate subcellular fractions show that the *esp2* mutant line accumulates glutelin precursors as long undigested peptide chains containing both subunits, located inside large protein storage vacuoles in the endosperm (Takemoto et al. 2002; Satoh-Cruz et al. 2010; Fukuda and Kumamaru 2019). The PDIL1-1 protein has high enzyme activity for both formation and reduction of disulfide bonds, compared with other rice PDIL proteins (Onda et al. 2011; Onda and Kobori 2014). Transcriptomics and proteomics approaches have shown that PDIL1-1 also controls the expression of other seed storage protein and starch biosynthesis genes (Han et al. 2012; Kim et al. 2012). Recently, additional biochemical analysis showed that PDIL1-1 creates disulfide bonds mediated by microRNA5144, and it directly interacts with the cysteine protease OsCP1 protein (Kim et al. 2012; Xia et al. 2018). The importance of PDIL1-1 in the biosynthesis and accumulation of grain components such as seed storage proteins and starches in rice endosperm is clear.

Rice with the *esp2* mutant allele has good flour and food processing characteristics suitable for making bread, although the original *esp2* mutant line has the disadvantage of quite low grain yield (Kawagoe 2010; Hori 2016). To develop novel rice cultivars showing good rice flour and food processing properties together with high grain yield, the *esp2* mutant was crossed with a high-eating-quality cultivar and with a high yield cultivar, and the resulting lines were evaluated.

## Materials and Methods

### Plant Materials

The *esp2* mutant EM747 was crossed with the high-eating-quality *japonica* rice cultivar, Koshihikari, to produce F_1_ plants. EM747 was induced by N-methyl-N-nitrosourea treatment of the *japonica* rice cultivar Taichung 65 (Takemoto et al. 2002). The EM747 plants have a nucleotide substitution at exon splicing sites in the *PDIL1-1* gene (Os11g0199200, LOC_Os11g09280), resulting in the nonfunctional gene allele. One F_6_ line was selected as Koshihikari *esp2,* using single seed descent and marker assisted selections from the F_2_ generation to develop an *esp2* mutant line with high agronomic performance. Next, Koshihikari *esp2* was crossed with the high yielding *indica* cultivar Oonari and self-pollinated to the BC_3_F_5_ generation, from which Oonari *esp2* was selected. Whole genome genotyping of Koshihikari *esp2* and Oonari *esp2* was carried out using the Fluidigm SNP genotyping platform (Fluidigm, San Francisco, CA, USA) with a set of 198 SNPs to detect polymorphisms between Koshihikari and EM747 and between Koshihikari and Oonari. Gene-specific DNA markers of 17 yield-related genes identified by Hori et al. (2021b) were used to genotype Koshihikari *esp2* and Oonari *esp2*. Koshihikari is a cultivar with high eating and cooking quality, which has been the most widely grown type in Japan for over 40 years (Kobayashi et al. 2018; Hori et al. 2021a) while Oonari is cultivar with high grain yield (Kobayashi et al. 2016). In this study, we tried to develop novel breeding lines showing high quality for rice flour and food processing characteristics together with high grain yield.

### Scoring Agronomic Traits

Rice cultivars and breeding lines were cultivated from 2013 to 2018, using six experimental fields in Japan. These were Tsukuba (36.02° N, 140.11° E) and Tsukubamirai (36.01° N, 140.02° E) at the Institute of Crop Science, NARO, Chikusei (36.30° N, 140.02° E) in the Oshima Nojo K.K., Fujieda (34.90° N, 138.28° E) at Shizuoka University, Kasai (34.88° N, 134.86° E) at Kobe University, and Chikugo (33.21° N, 130.49° E) at the Kyushu Okinawa Agricultural Research Center, NARO (Additional file 1: Fig. S2a). All experimental fields were within regions suitable for cultivation of Koshihikari and Oonari (Kobayashi et al. 2016, 2018). Koshihikari and Koshihikari *esp2* were grown at Tsukuba in 2016, 2017, and 2018, at Tsukubamirai in 2015 and 2016, and at Fujieda and Kasai in 2017. Oonari and Oonari *esp2* were cultivated at Tsukuba in 2016, and in all six experimental fields in 2017 and 2018. The planting density of each line was 20.0 individuals per m^2^ with two replications at all six experimental fields. Month-old seedlings were transplanted in mid-May and maturing plants were harvested in September at Tsukuba, Fujieda, and Kasai; seedlings were transplanted in early July and maturing plants were harvested in October at Tsukubamirai, Chikusei, and Chikugo. Agronomic traits were evaluated at each experimental field using standardized procedures.

### Evaluation of Grain Components

Total protein and amylose contents in matured grains were evaluated in each year from 2013 to 2018 using the methods of Hori et al. (2021b). Apparent amylose content was determined using an Auto Analyzer II (Bran + Luebbe, Norderstedt, Germany). Crude protein content was determined by the combustion method with an induction furnace at 900 °C (American Association of Cereal Chemists International, Approved Method 46-30.01). Measurement of low-molecular-weight compounds was carried out in 2015 and 2016, using the methods of Human Metabolome Technologies (Tsuruoka, Japan). Briefly, rice flours from each line were homogenized in 600 µL methanol containing 10 µM internal standards, mixed with 600 µL chloroform and 240 µL water, then centrifuged at 2300 × *g* for 5 min. The aqueous supernatant fraction was filtered and recovered into 50 µL of MilliQ water prior to metabolite analysis using capillary electrophoresis time of flight-mass spectrometry (CE-TOFMS) (Agilent Technologies, CA, USA) (Soga et al. 2004). Peaks detected in the CE-TOFMS analysis were extracted using automatic integration software (MasterHands ver. 2.17.1.11) and annotated with putative metabolites based on their migration in CE and m/z values.

### Evaluation of Grain Milling and Food Processing Properties

Grain milling characteristics and food processing properties were evaluated in Koshihikari and Koshihikari *esp2* from 2013 to 2016 and in Oonari and Oonari *esp2* in 2017 and 2018. Head brown rice grains of each line were polished to 90% of their original weight, to obtain polished (white) rice., and the polished rice yields for each line were recorded. The polished rice was milled by four methods: airflow wet grinding, airflow dry grinding, roll wet grinding, and roll dry grinding. Moisture, ash, damaged starch content, mean volume diameter (d50) of flour particles, and electricity consumption for milling were evaluated as rice flour characteristics, using the methods of Hayakawa et al. (2004) and Okunishi (2014). The mixing properties of rice flours were analyzed with a farinograph, using 500 BU as the standard. Rice breads were made from 20%, 30%, 50%, and 80% rice flours, with the balance wheat flour or appropriate gluten. The loaf volume, weight, and hardness after baking were evaluated. Sponge cakes were made with 100% rice flour, and their volume, weight, and center height after baking were evaluated. Rice noodles were made from 20 and 50% rice flours with the balance wheat flour or appropriate gluten, and their hardness, stickiness, and brittleness were evaluated by sensory eating tests after boiling noodles. Evaluations of food processing properties related to rice breads, cakes, and noodles were performed according to the methods of Hayakawa et al. (2004) and Okunishi (2014).

### Quantitative RT-PCR Analysis of Gene Expression

The causes of phenotypic differences in rice flour characteristics and food processing properties in Koshihikari, Koshihikari *esp2*, Oonari, and Oonari *esp2* were investigated by assessing the mRNA expression of 18 genes involved in seed storage protein synthesis, including the *PDIL* genes, the *Luminal binding protein* (*BiP*) gene family, and the *ER membrane-localized oxidoreductase 1* (*Ero1*) gene in immature rice grains at the grain filling stage. Total RNA was extracted from grains and from leaves at the grain filling stage of growth in 2018, using the RNeasy Plant Mini Kit (QIAGEN, Hilden, Germany) and primed with the oligo(dT)12-18 primer with SuperScript II reverse transcriptase (Invitrogen, MA, USA). cDNA corresponding to 50 ng of total RNA was used as the template for each SYBR Green–based PCR reaction, using gene-specific primers (Additional file 2: Table S1). Transcription levels of *PDI*-like genes, *BiP* family genes, *Ero1*, *Granule-bound starch synthase I* (*GBSSI*), *Starch synthase I* (*SSI*), *Starch synthase IIIa* (*SSIIIa*), and *Rice ubiquitin2* (*UBQ*) were determined according to the methods of Shibaya et al. (2016). Expression of the genes of interest was quantified relative to the expression of the *UBQ* gene. Gene expression data are presented as the means of at least three biological replicates, with three technical repeats for each biological replicate.

### SDS-PAGE Analysis of Seed Storage Proteins

Storage proteins from mature grain harvested in 2018 were extracted in the following series of elution buffers: 0.1 M NaCl, 70% ethanol, 0.1 M acetic acid, 2% SDS, and 2% SDS with 0.01 M DTT. Each elution sample was vigorously shaken for 30 min at 4 °C and then centrifuged at 15,000 × *g* for 5 min at 4 °C. Supernatants were used as loading samples for gel electrophoresis, and the precipitates (pellets) were dissolved in the next buffer solution in the series. Reducing and non-reducing SDS-PAGE analyses (with or without *2*-mercaptoethanol, respectively) were conducted on 12.5% polyacrylamide concentration gels. The total amount of protein was calculated as the sum of all proteins in each elution buffer, including supernatants and final precipitations in both reducing and non-reducing conditions, using the CN Coder MT700 mark2 (Yanako Technical Science, Tokyo, Japan).

## Results

### Development of the Koshihikari *esp2 *and Oonari *esp2* Lines

**Fig. 1:**
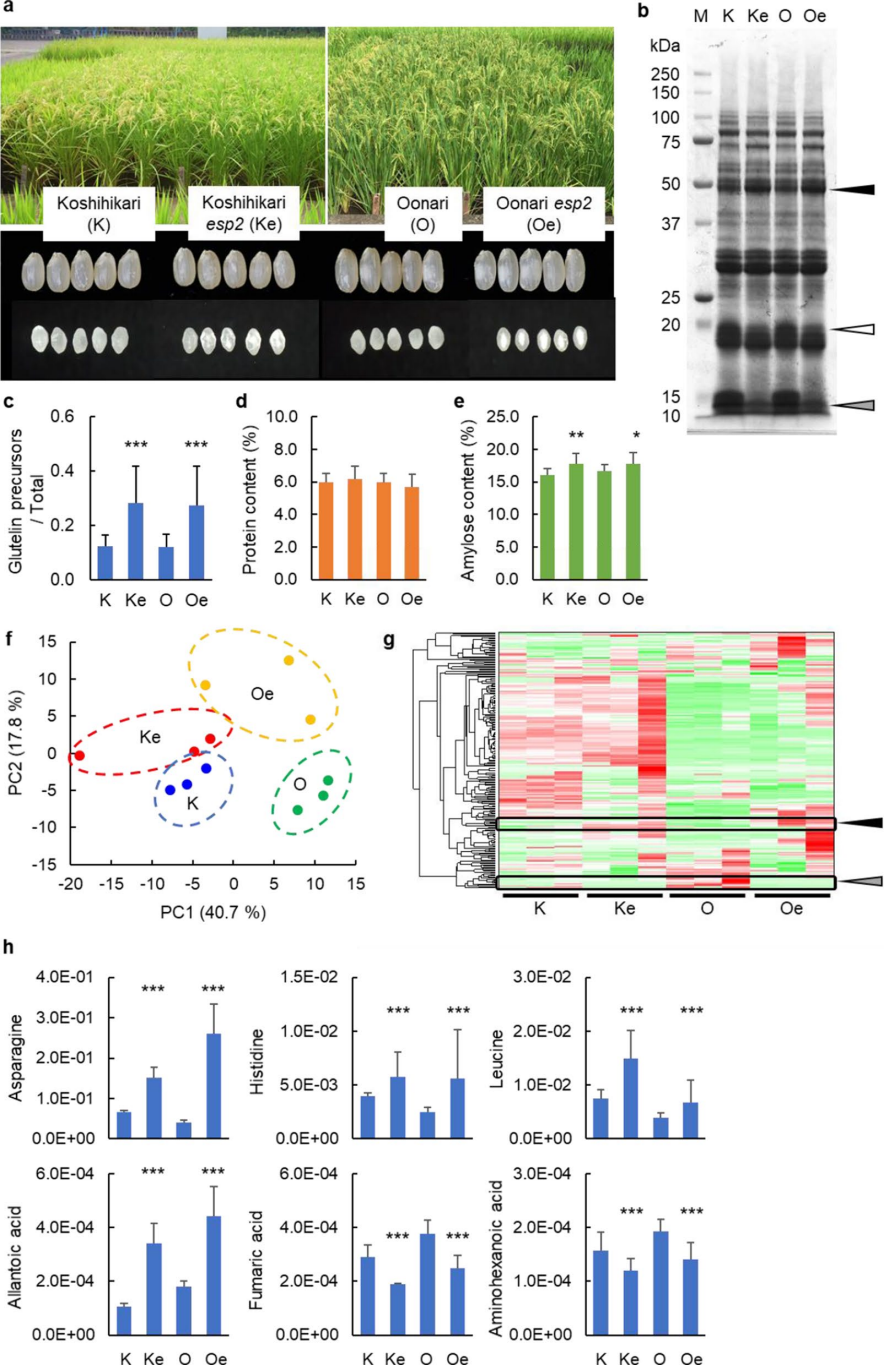
Phenotypic characteristics of rice from Koshihikari (K), Koshihikari *esp2* (Ke), Oonari (O), and Oonari *esp2* (Oe) lines. **a** Appearance of representative rice plants, matured grains and transections of grains in 2015. **b** Representative developed SDS-PAGE gel of seed storage proteins; black, white, and gray triangles indicate glutelin precursor, matured glutelin, and prolamin, respectively. **c** Glutelin precursor, **d** total protein, and **e** amylose content of mature rice grains; means of data from 2013 to 2018. **f** Principal component analysis, **g** hierarchical cluster analysis, and **h** relative area of low-molecular-weight metabolites in mature rice grains in 2015; black and gray triangles in **g** indicate increased and decreased levels of metabolites, respectively, in the *esp2* lines as compared with the respective parental lines. Asterisks indicate significant difference from the Koshihikari parental line at *P* < 0.05 (*), < 0.01 (**), and < 0.001 (***)

From the cross of the *esp2* mutant line EM747 with the high-eating-quality cultivar Koshihikari, 85 individual plants in the F_2_ generation showed a wide range of phenotypic variations for agronomic traits such as heading date and grain yield (Additional file 1: Fig. S1a–c). A DNA marker for the *esp2* mutation was identified in the F_2_, F_3_, F_4_, F_5_, and F_6_ lines (e.g., Additional file 1: Fig. S1d). Koshihikari *esp2* had good agronomic performance with early heading date and good grain yield (Fig. 1a, Additional file 1: Fig. S1b, c). Whole genome genotyping demonstrated that the Oonari *esp2* line developed from Koshihikari *esp2* has all 17 of the gene alleles from Oonari that are associated with high grain yield (Additional file 1: Fig. S1f). This confirmed that the marker-assisted selection successfully introduced the *esp2* mutation into both breeding lines.

In 2016, Koshihikari *esp2* had earlier heading date (3 August vs. 19 August), increased unhulled grain weight (22.0 vs. 14.2 g/plant), and increased head brown grain weight (18.3 vs. 12.1 g/plant) compared with the original *esp2* mutant line EM747 (Table 1). Interestingly, several agronomic traits in Koshihikari *esp2* were different from those in the Koshihikari parent line (Table 1). For example, in 2016 the heading dates were similar in Koshihikari *esp2* and Koshihikari (3 and 1 August, respectively), but significant decreases were observed in plant height (65.5 vs. 73.3 cm), number of tillers per plant (12.0 vs. 13.8), culm length (88.7 vs. 93.1 cm), unhulled grain weight (22.0 vs. 30.5 g/plant), and head brown rice grain weight (18.3 vs. 25.0 g/plant). Similar differences were observed in other years and at other field locations (Table 1, Additional file 1: Fig. S2, Additional file 2: Tables S2, S3). Oonari *esp2* had high unhulled grain weight and head brown grain weight (e.g., 32.3 g/plant and 27.4 g/plant, respectively, in 2016; Table 1), but these were not significantly different from Oonari. Oonari *esp2* and Oonari were also similar in other agronomic traits including heading date, plant height, culm length, and number of tillers (Table 1). In summary, incorporation of the *esp2* mutant allele changed several agronomic traits in the Koshihikari genetic background, but not in Oonari.

**Table 1:** Agronomic characteristics of Koshihikari, Koshihikari *esp2*, Oonari, and Oonari *esp2* lines grown in the Tsukuba field in 2016, 2017, and 2018

		Maxumum tiller number stage				Heading date (day)	Maturation stage									
Year	Line	Plant height (cm)	Leaf color (SPAD)	Leaf width (cm)	No. of tillers (No./plant)		Culm length (cm)	Panicle length (cm)	No. of panicles (No./plant)	Leaf color (SPAD)	Unhulled rice weight (g/plant)	Head brown rice weight (g/plant)	Ratio of head brown rice weight (%)	Polished rice yield (%)	Leaf blast resistance (0–9)	Panicle blast resistance (0–9)
2016	Koshihikari	73.3	40.2	11.2	13.8	8/1	93.1	20.3	9.8	29.3	30.5	25.0	100	91.9	7.8	9.0
	Koshihikari *esp2*	65.5	37.9	10.1	12.0	8/3	88.7	21.9	9.9	32.2	22.0	18.3	73	89.6	8.0	4.4
	Oonari	72.7	35.3	11.3	15.7	8/6	74.8	24.5	8.4	28.7	34.4	30.3	121	90.0	0.0	–
	Oonari *esp2*	66.5	35.7	11.4	17.6	8/8	76.1	26.2	8.4	31.8	32.3	27.4	110	90.1	0.0	–
	EM747 (Taichung 65 *esp2*)	–	–	–	–	8/19	–	–	–	–	14.2	12.1	48	–	6.2	5.3
2017	Koshihikari	76.3	40.5	11.6	14.0	7/31	85.4	21.0	10.6	28.9	26.9	21.5	100	90.1	–	–
	Koshihikari *esp2*	68.7	41.2	9.4	13.4	8/2	76.8	20.4	8.8	31.1	21.8	17.7	83	88.7	–	–
	Oonari	82.2	33.5	11.2	13.4	8/7	78.6	21.8	9.0	29.9	33.2	26.0	121	90.7	–	–
	Oonari *esp2*	83.6	35.6	10.2	13.7	8/9	76.4	23.8	9.4	32.7	31.0	24.2	112	89.1	–	–
2018	Koshihikari	75.9	39.5	11.0	15.6	7/27	96.2	19.7	12.9	30.5	31.5	26.3	100	90.6	–	–
	Koshihikari *esp2*	67.7	40.3	9.2	14.6	7/31	85.4	22.3	14.7	25.1	22.9	19.6	75	90.2	–	–
	Oonari	89.2	34.9	14.6	33.2	8/3	71.5	26.2	14.2	28.9	41.3	30.4	116	90.7	–	–
	Oonari *esp2*	90.4	37.2	15.4	31.0	8/5	62.9	27.4	13.6	33.0	33.7	28.5	108	88.7	–	–

### Grain Components of the Koshihikari and Oonari *esp2* Lines

Koshihikari *esp2* and Oonari *esp2* had translucent grains with some chalkiness (Fig. 1a; Hori 2018). Both *esp2* lines accumulated more than twice the quantity of glutelin precursors in the matured grain as was observed in the respective parental lines (Fig. 1b, c). Accumulation of glutelin precursors is one of the representative phenotypes of the *esp2* and other seed storage protein mutations associated with transport of proteins from ER to Golgi and protein bodies (PBI and PBII) (Takemoto et al. 2002; Fukuda and Kumamaru 2019). The total protein content of matured grain was similar in the two *esp2* lines, but the amylose content was about 2% higher in the breeding lines than in the parental lines (Fig. 1d, e). Metabolome analysis with CE-TOFMS detected a total of 213 compounds in Koshihikari, Koshihikari *esp2*, Oonari, and Oonari *esp2* in 2015 and 240 compounds in 2016 (Fig. 1g, Additional file 1: Fig. S3, Additional file 2: Table S4). Principal component hierarchical cluster analysis of the 2015 data clearly distinguished the different cultivars as the first principal component and the presence or absence of the *esp2* mutation as the second principal component (Fig. 1f). Koshihikari *esp2* and Oonari *esp2* had increased content of several amino acids and organic acids such as asparagine, histidine, leucine, and allantoic acid compared with the respective parental lines, but decreased content of several fatty acids such as fumaric acid and 6-aminohexanoic acid in 2015 (Fig. 1h) and 2016 (Additional file 1: Fig. S3, Additional file 2: Table S4). Saccharide contents, including glucose, sucrose, and fructose, were not significantly different between Koshihikari *esp2* and Koshihikari or between Oonari *esp2* and Oonari (data not presented). These observations indicate that the alterations of grain components attributable to introduction of the *esp2* mutation were similar in the Koshihikari and Oonari genetic backgrounds.

### Grain Milling and Food Processing Properties in the Koshihikari and Oonari *esp2* Lines

Several starch and seed storage protein mutants significantly decrease polished (white) rice yields due to high incidence of chalky and soft grains (Ashida et al. 2009; Mo and Jeung 2020). However, in the work reported here, the Koshihikari *esp2* and Oonari *esp2* lines showed the same proportion of polished rice (approximately 90.0%) as in their respective parental lines during three years of testing (Table 1). This suggests that the *esp2* mutation maintained polished rice yield, unlike other reported starch and seed storage protein mutant lines.

**Fig. 2:**
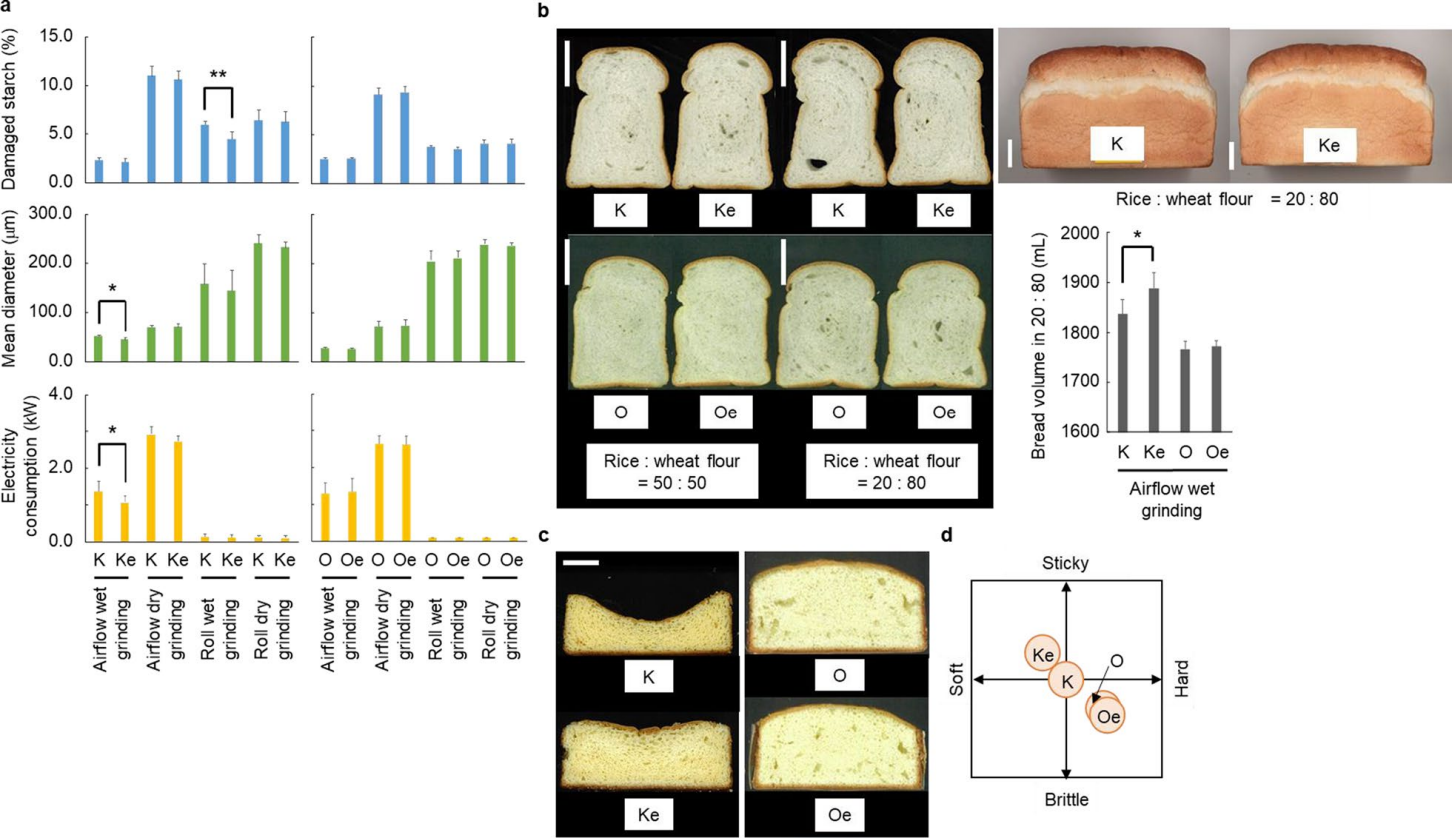
Rice flour milling and food processing characteristics. **a** Damaged starch content, starch granule diameter, and electricity consumption; means of data from 2013 to 2018. **b** Bread-making properties; scale bars indicate 5 cm. **c** Sponge cake properties; scale bars indicate 1 cm. **d** Sensory testing of rice noodles. Asterisks indicate significant difference between the parental and the *esp2* lines at *P* < 0.05 (*) and < 0.01 (**). Cultivar abbreviations as in Fig. 1

Among the four milling methods used in all six years of the work reported here, the airflow wet grinding method produced the smallest mean diameter of rice flours with the lowest content of damaged starch (Fig. 2a, Additional file 2: Table S5). In 2013, Koshihikari *esp2* had significantly smaller diameter of rice flour (38.6 µm) and decreased electricity consumption (1.0 kW) in the airflow wet grinding method and lower damaged starch content (5.4%) in the roll wet grinding method, as compared with Koshihikari (51.1 µm, 1.5 kW, and 6.4%, respectively). In contrast, Oonari *esp2* scores for percent damaged starch, mean flour diameter, and electricity consumption were not significantly different from Oonari in all four milling methods.

Rice flours milled by the airflow wet grinding method were chosen to evaluate in the making of bread, sponge cake, and noodles because this method produced the highest quality flour (i.e., lowest mean diameter and damaged starch content, Fig. 2a). Rice breads made with Koshihikari *esp2* flour from 2013 to 2016 had larger volumes in all levels of rice:wheat content, compared to breads made with Koshihikari flour (Fig. 2b, Additional file 2: Table S5). For example, in 2013, volumes of rice breads made with 20% rice flour by roll wet grinding method were 1879.0 and 1854.6 mL for Koshihikari *esp2* and Koshihikari, respectively. Rice breads made from Oonari *esp2* and Oonari flours had similar volumes in all ratios of rice flour to wheat flour (e.g., volumes of 1900.1 and 1895.6 mL, respectively, at 20% rice flour in 2017), but these breads did not rise as high as breads made with the Koshihikari *esp2* and Koshihikari flours (Fig. 2b, Additional file 2: Table S5). Sponge cakes made from Koshihikari *esp2* rice flour had larger volume than those made of flour from the parental Koshihikari line (1346.7 vs. 1306.0 mL, respectively, in 2013; Fig. 2c, Additional file 2: Table S5). Sponge cakes made using the Oonari *esp2* and Oonari flours had similar heights and volumes, (e.g., 1262.4 and 1271.1 mL, respectively, in 2017), but both were smaller than cakes made from the Koshihikari *esp2* and Koshihikari flours (Fig. 2c, Additional file 2: Table S5). In sensory tests of rice noodles made from 50% rice flour, the Koshihikari *esp2* noodles had softer texture and greater stickiness than Koshihikari noodles (Fig. 2d). Rice noodles made from Oonari *esp2* and Oonari flours had similar texture, but they were more brittle and harder than the noodles made from Koshihikari and Koshihikari *esp2* flours (Fig. 2d).

### Differential Gene Expression of the *esp2* Gene in Different Genetic Backgrounds

**Fig. 3:**
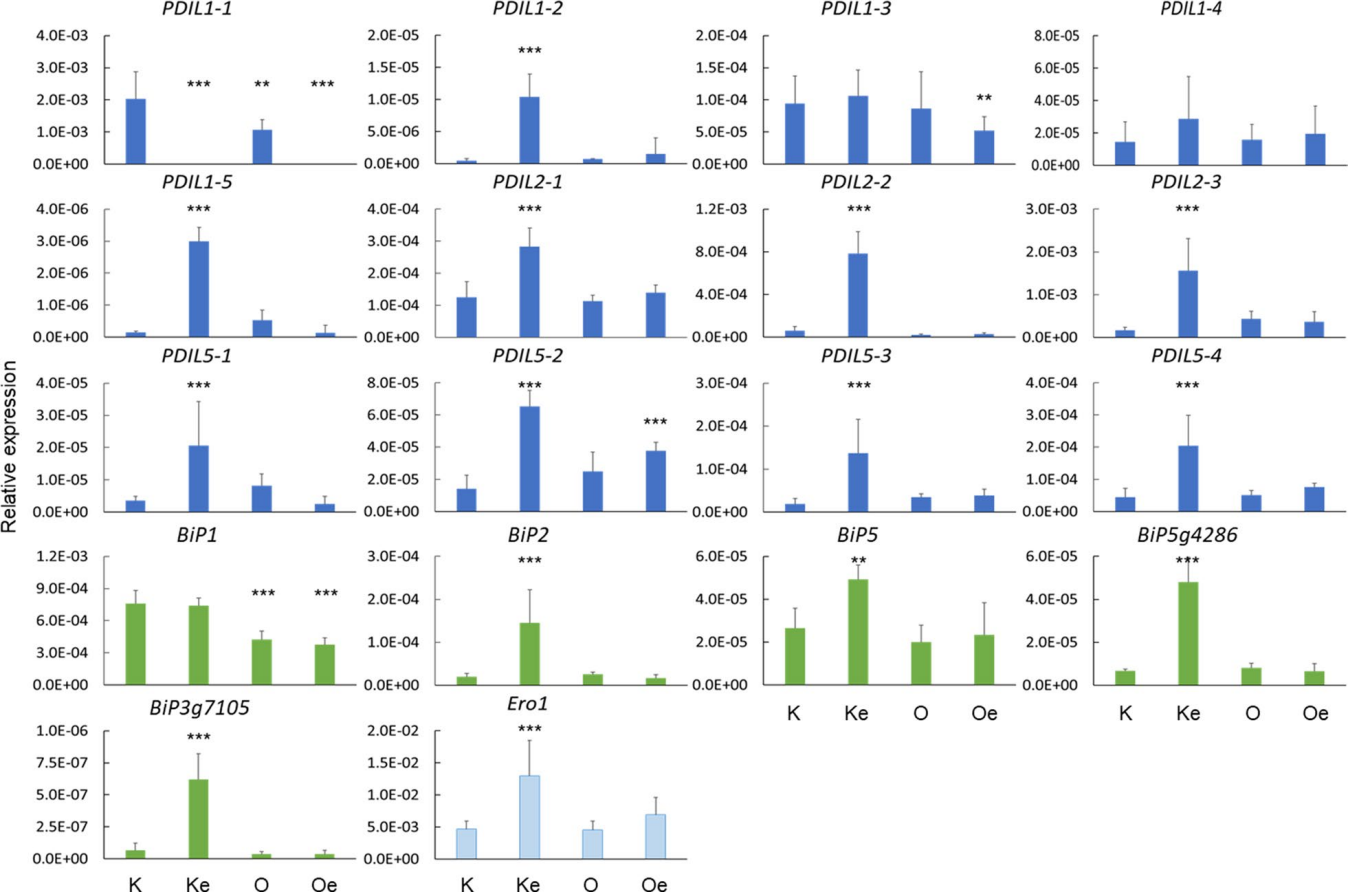
Expression of seed storage protein biosynthesis genes in immature grain, for twelve *PDIL*, one *Ero*, and five *Bip* genes, relative to expression of *UBQ*. Asterisks indicate significant difference from the Koshihikari parental line at *P* < 0.01 (**) and < 0.001 (***); cultivar abbreviations as in Fig. 1

The *PDIL* and *BiP* genes code for the two main classes of chaperone involved in the folding of seed storage proteins in the ER lumen; the *Ero1* gene product donates disulfide bonds to the *PDIL* gene products (Onda et al. 2009). In the work reported here, expression of the *PDIL1-1* gene was absent from maturing endosperms in Koshihikari *esp2* and Oonari *esp2* (Fig. 3), which can be attributed to the deficient mutation of this gene. Interestingly, expression of mRNA in nine other *PDIL* genes, four of the *BiP* genes, and the *Ero1* gene was significantly higher (*P* < 0.01 or better) in Koshihikari *esp2* than in the Koshihikari parental line. Expression of the remaining genes was not significantly different in Koshihikari *esp2* and Koshihikari. In contrast, in the Oonari *esp2* line, expression of mRNA differed from the Oonari parental line only in *PDIL5-2* (higher*)* and in *PDIL1-1* and *PDIL1-3* (lower; Fig. 3). Expression in Oonari *esp2* of the remaining 15 genes was similar to both Oonari and Koshihikari.

In the leaf tissue of rice plants at the grain filling stage, gene expression of *PDIL1-1* was absent in the breeding lines of both cultivars (Additional file 1: Fig. S4), as was observed in immature rice grains. In addition, seven other genes had significantly different (*P* < 0.05) expression in the leaves of Koshihikari *esp2* and Koshihikari, and two genes had different expression in Oonari *esp2* and Oonari.

Expression of the *GBSSI* starch (amylose and amylopectin) biosynthesis genes increased, but expression of *SSI* and *SSIIIa* genes was unchanged in the endosperm of Koshihikari *esp2* and Oonari *esp2* in comparison with the Koshihikari parental line (Additional file 1: Fig. S5). *GBSSI* is associated with the biosynthesis of amylose and extra-long chain amylopectin, and *SSI* and *SSIIIa* are associated with biosynthesis of the side chains of amylopectin. These three genes are highly expressed in rice endosperm at the grain filling stage compared with other starch biosynthesis genes.

### Composition of Seed Storage Proteins in the Two Genetic Backgrounds

**Fig. 4:**
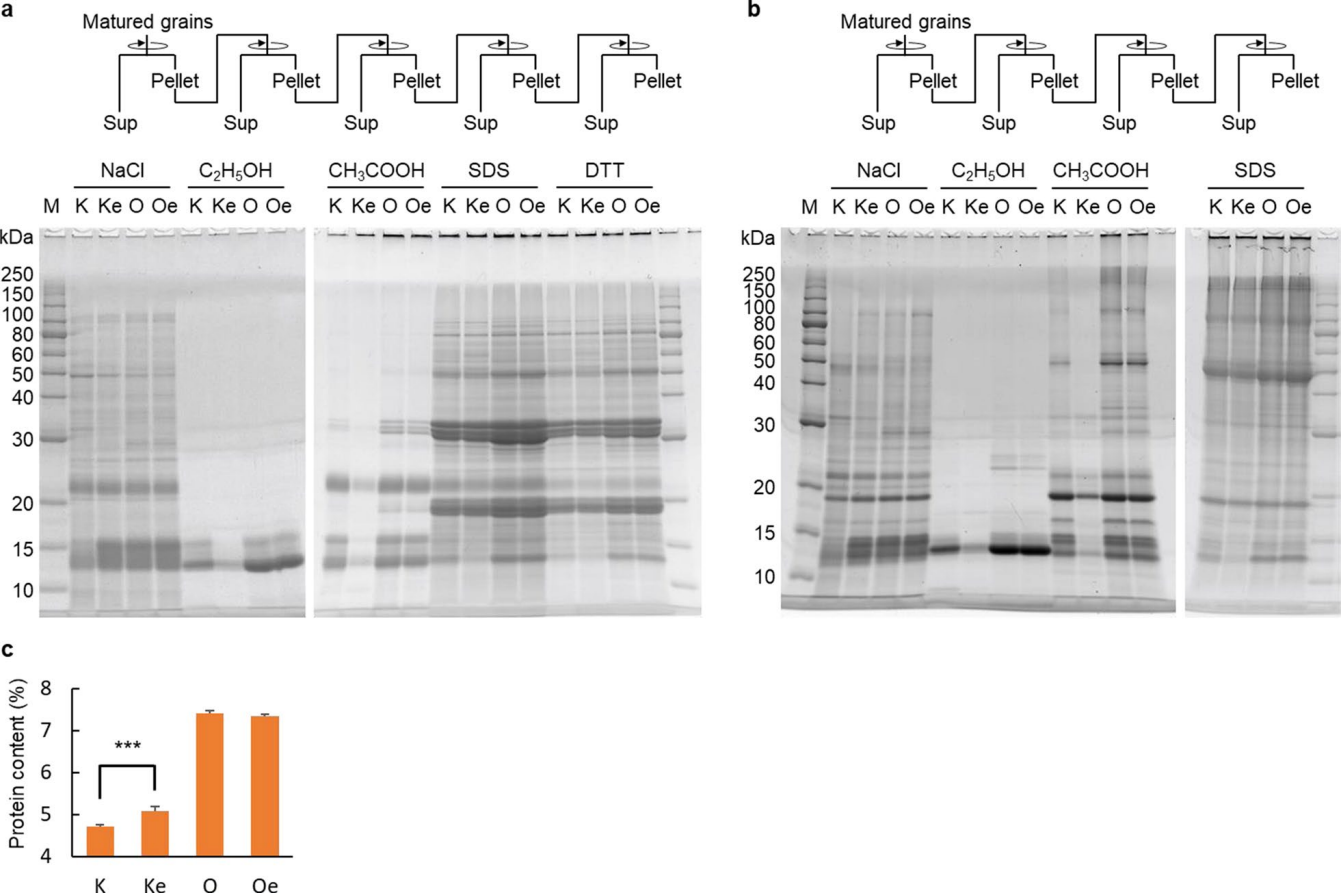
SDS-PAGE analysis of seed storage proteins of mature rice grains, in centrifugation supernatants after serial treatment with NaCl, C_2_H_5_OH, CH_3_COOH, SDS, and SDS with DTT. **a** Under reducing conditions, and **b** non-reducing conditions (but no SDS-DTT step). **c** Total protein content, sum of all supernatants, and final precipitations. Cultivar abbreviations as in Fig. 1

Reducing and non-reducing gel electrophoresis of seed storage protein conformations and structures, including the disulfide bond content of rice endosperm, helped elucidate some of the differences in expression of the seed storage protein synthesis genes. In comparison with Koshihikari, several protein bands from 40 to 10 kDa were decreased in Koshihikari *esp2* in the C_2_H_5_OH and CH_3_COOH fractions under reducing conditions (Fig. 4a). In non-reducing conditions in the C_2_H_5_OH, CH_3_COOH, and SDS fractions, several protein bands from 250 to 10 kDa were decreased in Koshihikari *esp2* compared with the other three lines (Fig. 4b). Koshihikari *esp2* had significantly more (*P* < 0.01) total protein than the Koshihikari parental line (Fig. 4c). In contrast, Oonari and Oonari *esp2* had similar protein band patterns and total protein content.

## Discussion

This study indicates that certain genetic backgrounds negated phenotypic and gene expression changes attributable to the mutant allele in another background. We developed two rice breeding lines by introducing the *PDIL1-1* (*esp2*) mutation into two different genetic backgrounds, a high-eating-quality cultivar, Koshihikari, and a high-yielding cultivar, Oonari. A DNA marker for genotyping the *PDIL1-1* mutation was useful for efficient marker-assisted selection. Both *esp2* lines accumulated higher quantities of glutelin precursors and had higher amylose contents, with similar composition of low-molecular-weight metabolites such as amino acids, organic acids, and fatty acids in comparison with the respective parental lines. However, there were significant differences in agronomic traits, rice flour characteristics, and food processing properties between the two *esp2* lines. The Koshihikari *esp2* line had good rice flour characteristics and food processing properties, but decreased plant height and grain yield compared with Koshihikari. In the Oonari *esp2* line, rice flour characteristics, food processing properties, and grain yield all remained similar to the parental Oonari line. These phenotypic differences are likely attributable to formation of high-molecular-weight protein complexes in the endosperm caused by observed increases in mRNA expression of the seed storage protein synthesis genes *PDIL, BiP*, and *Ero1* in Koshihikari *esp2*, but not seen in Oonari *esp2.*

Previous studies regarding *PDIL1-1* (*esp2*) have used knockout mutant lines and knockdown (RNAi) transgenic lines in the genetic backgrounds of *japonica* rice cultivars Kinmaze, Taichung 65, Nipponbare, Yukihikari, Dongjin, and Zhonghua11 (Takemoto et al. 2002; Onda et al. 2009; Satoh-Cruz et al. 2010; Onda et al. 2011; Han et al. 2012; Kim et al. 2012; Onda and Kobori 2014; Xia et al. 2018). These studies report that nonfunctional and decreased *PDIL1-1* alleles result in similar phenotypes with altered stacking of seed storage proteins within the ER and decreases of enzymatic activity in the formation and reduction of disulfide bonds. Koshihikari is also a *japonica* rice cultivar and has been the most widely grown type in Japan for over 40 years (Kobayashi et al. 2018). Oonari is an *indica* rice cultivar derived from a cross between Milyang 42 and Milyang 25 (Kobayashi et al. 2016). According to the TASUKE database (Kumagai et al. 2019), both Koshihikari and Oonari have only one *PDIL1-1* gene in their genome sequences. In the work reported here, the mutant *PDIL1-1* gene changed the mRNA expression of several *PDIL* and *BiP* genes and the *Ero1* gene in the immature endosperm of the Koshihikari *esp2* line, but not in immature endosperm of Oonari *esp2.* This suggests that phenotypic expression of the *PDIL1-1* (*esp2*) mutant allele may have been unattainable in Oonari due to a lack of expression of other *PDIL* and/or the *BiP* and *Ero1* genes.

The rice genome contains 12 *PDIL* genes (Houston et al. 2005). Among these, seven of them (*PDIL1-1*, *1-2*, *1-3*, *1-4*, *2-1*, *2-2*, and *2-3*) contain two redox active cysteine pair domains. The redox active cysteine pair domain has a catalytic activity function for both formation and reduction of disulfide bonds. The PDIL1-1 protein has higher catalytic activity for disulfide bond formation than other PDIL proteins, such as PDIL1-4 and PDIL2-3, as shown by the oxidative RNase refolding assay (Onda and Kobori 2014). The *PDIL1-1* and *Ero1* genes function on the same electron transfer pathway for intra-molecular disulfide bond formation between acid- and alkali-soluble glutelin subunits (Onda et al. 2009). Expressions of several *BiP* genes are increased by ER stress and an unfolded protein response increase expression of *BiP* genes when deficient mutations of the *PDIL1-1* and *Ero1* genes are present (Onda et al. 2009, 2011; Satoh-Cruz et al. 2010; Han et al. 2012; Kim et al. 2012). In the study reported here, parts of the regulatory pathway via the *PDIL1-1* gene were altered in the Koshihikari *esp2* plants, as seen in those previous studies. Conversely, in the Oonari *esp2* plants, no significant differences in mRNA expression were observed in many of the *PDIL*, *BiP*, and *Ero1* genes. This suggests that there are unknown genes responsible for the expression of phenotypes attributable to the *esp2* mutation. Further experimental studies are necessary to identify the unknown genes associated with these differences between Koshihikari and Oonari.

In wheat cultivars, genotypes that code for high-molecular-weight glutenin subunits (HMW-GS) usually predict good bread- and dough-making quality based on the presence of individual glutenin subunits (Branlard et al. 2001). However, wheat cultivars having the same HMW-GS genotypes have also shown large phenotypic variations in bread-making scores. The PDI proteins are believed to be among several factors with the potential to change the bread-making properties of wheat (Demska et al. 2018). In fact, addition of recombinant PDI and Ero1 proteins to wheat flours increased disulfide bond formation in the bread dough, improving bread-making properties (Noguchi et al. 2015, 2016). In this study, Koshihikari *esp2* had high bread-making scores even though this line has the nonfunctional *PDIL1-1* gene allele. PDIL1-1 functions in the formation of intra-molecular disulfide bonds between two glutelin subunits. Improved bread-making properties in Koshihikari *esp2* implies complementary effects of the *PDIL1-1* gene with other *PDIL* and *BiP* genes to increase inter-molecular disulfide bonds among seed storage proteins, forming high-molecular-weight protein complexes.

High-yield rice cultivars are needed to produce large amounts of food for a growing global population. At the same time, income growth and urbanization will lead to increased consumer demands for high eating quality in various cereal foods including rice (Ito et al. 1989; Hori and Yano 2013; Hori 2018; Sharma et al. 2018; Hori et al. 2021b). The wide prevalence of celiac disease and wheat allergies is also increasing the demand for gluten-free foods (Ashida et al. 2009; Yano 2012; Montemurro et al. 2021). Therefore, it is important to develop high-yield rice cultivars for making rice flour foods including breads, noodles, and cakes.

## Conclusions

Koshihikari and Oonari breeding lines with the *esp2* mutant allele accumulated glutelin precursors and several low-molecular-weight metabolites such as amino acids and organic acids in their endosperm. The Koshihikari *esp2* line showed the good flour characteristics and food processing properties than the Koshihikari parental line, whereas the Oonari *esp2* line did not show a significant improvement in these traits compared to Oonari. Compared with the parental line, the endosperm of Koshihikari *esp2* had elevated mRNA expression of the *PDI* and *BiP* gene families and formed high-molecular-weight protein complexes, but these alterations were not observed in Oonari *esp2* endosperm. These results suggest that different genetic backgrounds can alter the phenotypic expression of a gene for rice flour characteristics and food processing properties, even when the same mutant gene allele is introduced to both genotypes. The work reported here has elucidated a part of the molecular basis of these phenotypic differences between breeding lines with the same mutant alleles in different genetic backgrounds. This may provide novel insights for further breeding efforts to introduce mutant gene alleles.

## Supplementary Information

**Additional file 1: Fig. S1.** Development of rice breeding lines introducing the *esp2* mutation. **a** Pedigree of Koshihikari *esp2* and Oonari *esp2*. **b** Days to heading in 85 F_2_ individuals derived from a cross between EM747 and Koshihikari. **c** Diversity of panicle size phenotypes in 85 F_2_ individuals from the cross of EM747 and Koshihikari. **d** DNA marker for detecting the *esp2* mutant allele. **e** Whole genome genotype of Koshihikari *esp2*. **f** Whole genome genotype and positions of *PDIL* and 17 yield-related genes in Oonari *esp2*. K, Koshihikari; Ke, Koshihikari *esp2*; O, Oonari; Oe, Oonari *esp2*. **Fig. S2.** Grain yield of Koshihikari *esp2* and Oonari *esp2* in 2017 and 2018. **a** Locations of the six experimental fields in Japan. **b** Head brown rice weight of Koshihikari and Koshihikari *esp2* at three locations in 2017. **c** Head brown rice weight in Oonari and Oonari *esp2* at six locations in 2017 and 2018. Asterisks indicate significant difference from the Koshihikari parental line at *P* < 0.001. **Fig. S3.** Results of metabolome analysis of matured grains in 2016. **a** Principal component analysis of metabolites (*n* = 3). **b** Hierarchical cluster analysis of metabolites. Black and gray triangles indicate increased and decreased compounds, respectively, in the *esp2* lines in comparison with the respective parental lines. **c** Quantities of low-molecular-weight metabolites. Asterisks indicate significant difference from the Koshihikari parental line at *P* < 0.05 (*), < 0.01 (**) and < 0.001 (***). Cultivar abbreviations as in Additional file 1: Fig. S1. **Fig. S4.** Expression of seed storage protein biosynthesis genes in rice leaves at the grain filling stage. The expression of twelve *PDIL*, one *Ero*, and five *Bip* family genes relative to the expression of *UBQ*. Asterisks indicate significant difference from the Koshihikari parental line at *P* < 0.05 (*), < 0.01 (**), and < 0.001 (***). Cultivar abbreviations as in Additional file 1: Fig. S1. **Fig. S5.** Expression of starch biosynthesis genes *GBSSI*, *SSI*, and *SSIIIa* in rice immature grain and in leaves at the grain filling stage. Gene expression is relative to expression of the *UBQ*. Asterisks indicate significant difference from Koshihikari at *P* < 0.05 (*), < 0.01 (**), and < 0.001 (***). Cultivar abbreviations as in Additional file 1: Fig. S1.

**Additional file 2: Table S1.** Primer sequences used in this study. **Table S2.** Agronomic characteristics of Koshihikari, Koshihikari *esp2*, Oonari, and Oonari *esp2* lines grown in the Chikusei, Fujieda, Kasai, and Chikugo fields in 2017 and 2018. **Table S3.** Agronomic characteristics of Koshihikari, Koshihikari *esp2*, Oonari, and Oonari *esp2* lines grown in the Tsukubamirai field in 2015, 2016, 2017, and 2018. **Table S4.** Low-molecular-weight metabolites in Koshihikari, Koshihikari *esp2*, Oonari, and Oonari *esp2* lines in 2015 and 2016. **Table S5.** Rice flour milling and food processing characteristics of rice from the Koshihikari, Koshihikari *esp2*, Oonari, and Oonari *esp2* lines from 2013 to 2018.

## Data Availability

All datasets supporting the conclusions of this article are included in the article and Additional files 1, 2.

## Abbreviations

BiP: Luminal binding protein
DTT: Dithiothreitol
ER: Endoplasmic reticulum
Ero1: ER membrane-localized oxidoreductase 1
PBII: Protein body II
PDI: Protein disulfide isomerase
SDS-PAGE: Sodium dodecyl sulfate–polyacrylamide gel electrophoresis
SNP: Single nucleotide polymorphism
