# Supplementary material for: Genetic Background Negates Improvements in Rice Flour Characteristics and Food Processing Properties Caused by a Mutant Allele of the PDIL1-1 Seed Storage Protein Gene
Source: Rice (N Y). 2022 Mar 5;15:13. doi: 10.1186/s12284-022-00560-w (PMC8898210; doi:10.1186/s12284-022-00560-w)
Supplement: Supplementary file 1 — Additional file 1: Fig. S1. Development of rice breeding lines introducing the esp2 mutation. a Pedigree of Koshihikari esp2 and Oonari esp2. b Days to heading in 85 F2 individuals derived from a cross between EM747 and Koshihikari. c Diversity of panicle size phenotypes in 85 F2 individuals from the cross of EM747 and Koshihikari. d DNA marker for detecting the esp2 mutant allele. e Whole genome genotype of Koshihikari esp2. f Whole genome genotype and positions of PDIL and 17 yield-related genes in Oonari esp2. K, Koshihikari; Ke, Koshihikari esp2; O, Oonari; Oe, Oonari esp2. Fig. S2. Grain yield of Koshihikari esp2 and Oonari esp2 in 2017 and 2018. a Locations of the six experimental fields in Japan. b Head brown rice weight of Koshihikari and Koshihikari esp2 at three locations in 2017. c Head brown rice weight in Oonari and Oonari esp2 at six locations in 2017 and 2018. Asterisks indicate significant difference from the Koshihikari parental line at P < 0.001. Fig. S3. Results of metabolome analysis of matured grains in 2016. a Principal component analysis of metabolites (n = 3). b Hierarchical cluster analysis of metabolites. Black and gray triangles indicate increased and decreased compounds, respectively, in the esp2 lines in comparison with the respective parental lines. c Quantities of low-molecular-weight metabolites. Asterisks indicate significant difference from the Koshihikari parental line at P < 0.05 (*), < 0.01 (**) and < 0.001 (***). Cultivar abbreviations as in Additional file 1: Fig. S1. Fig. S4. Expression of seed storage protein biosynthesis genes in rice leaves at the grain filling stage. The expression of twelve PDIL, one Ero, and five Bip family genes relative to the expression of UBQ. Asterisks indicate significant difference from the Koshihikari parental line at P < 0.05 (*), < 0.01 (**), and < 0.001 (***). Cultivar abbreviations as in Additional file 1: Fig. S1. Fig. S5. Expression of starch biosynthesis genes GBSSI, SSI, and SSIIIa in ric [file 12284_2022_560_MOESM1_ESM.pptx]

## Slide 1
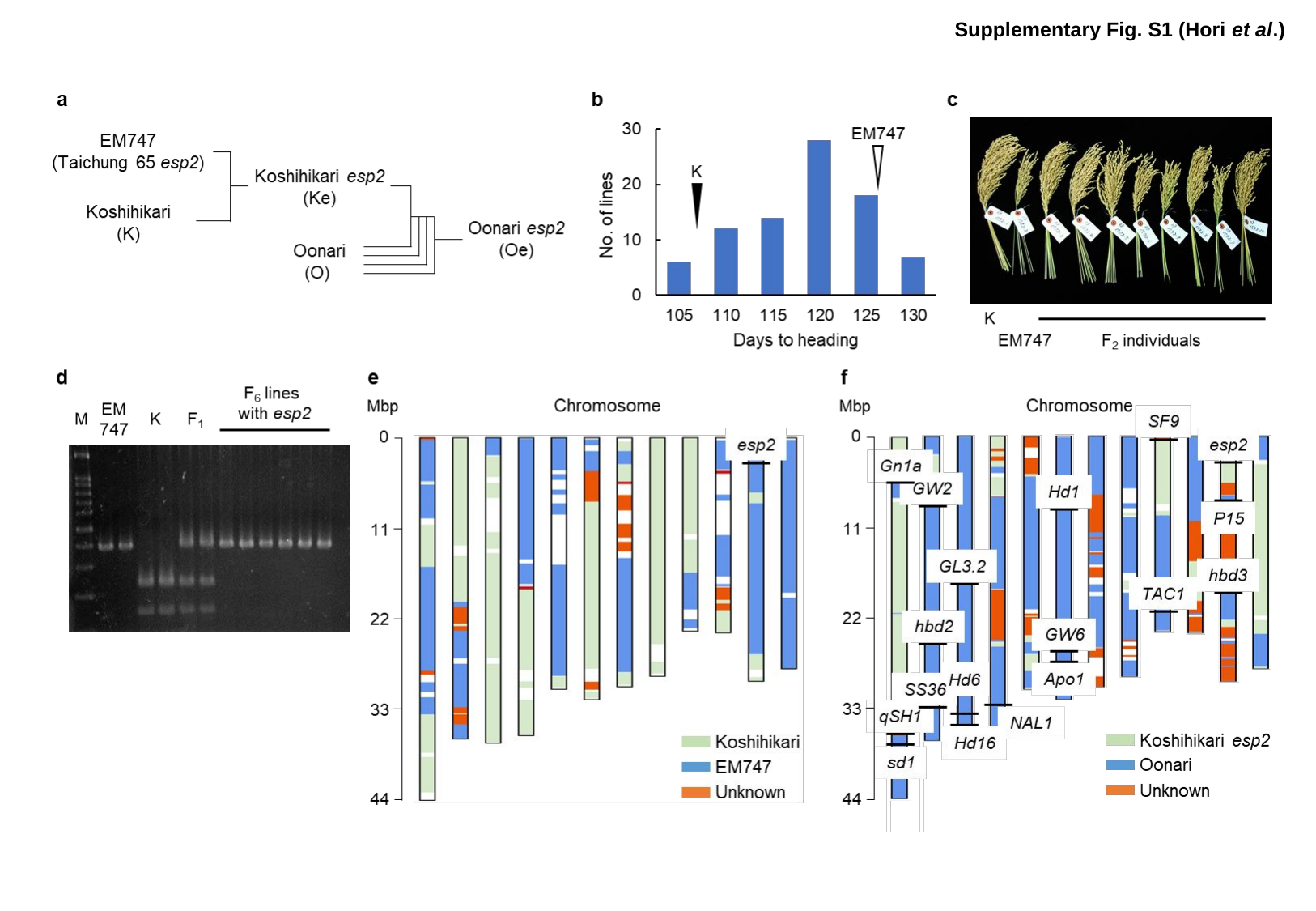

Supplementary Fig. S1 (Hori et al.)

## Slide 2
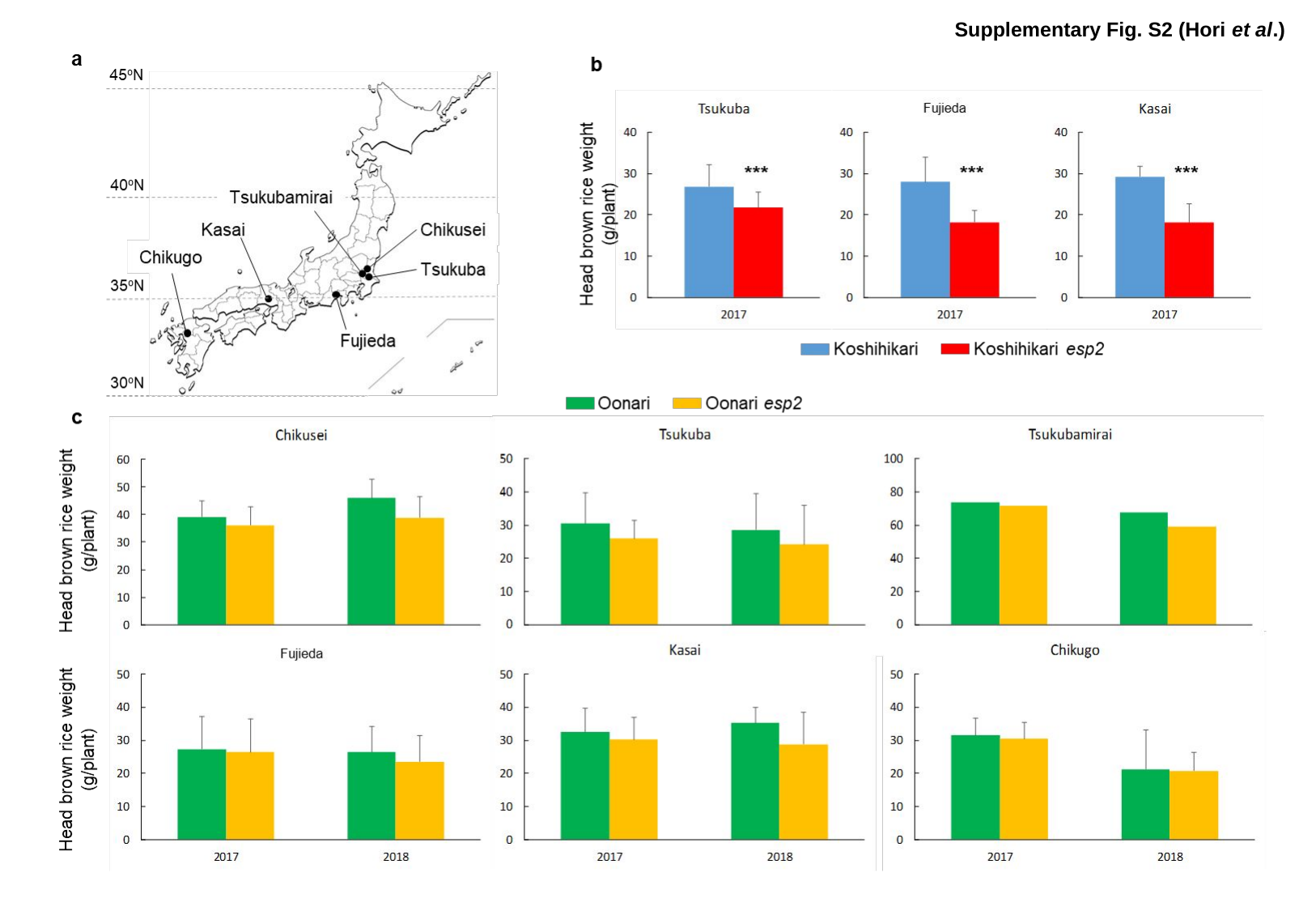

Supplementary Fig. S2 (Hori et al.)

## Slide 3
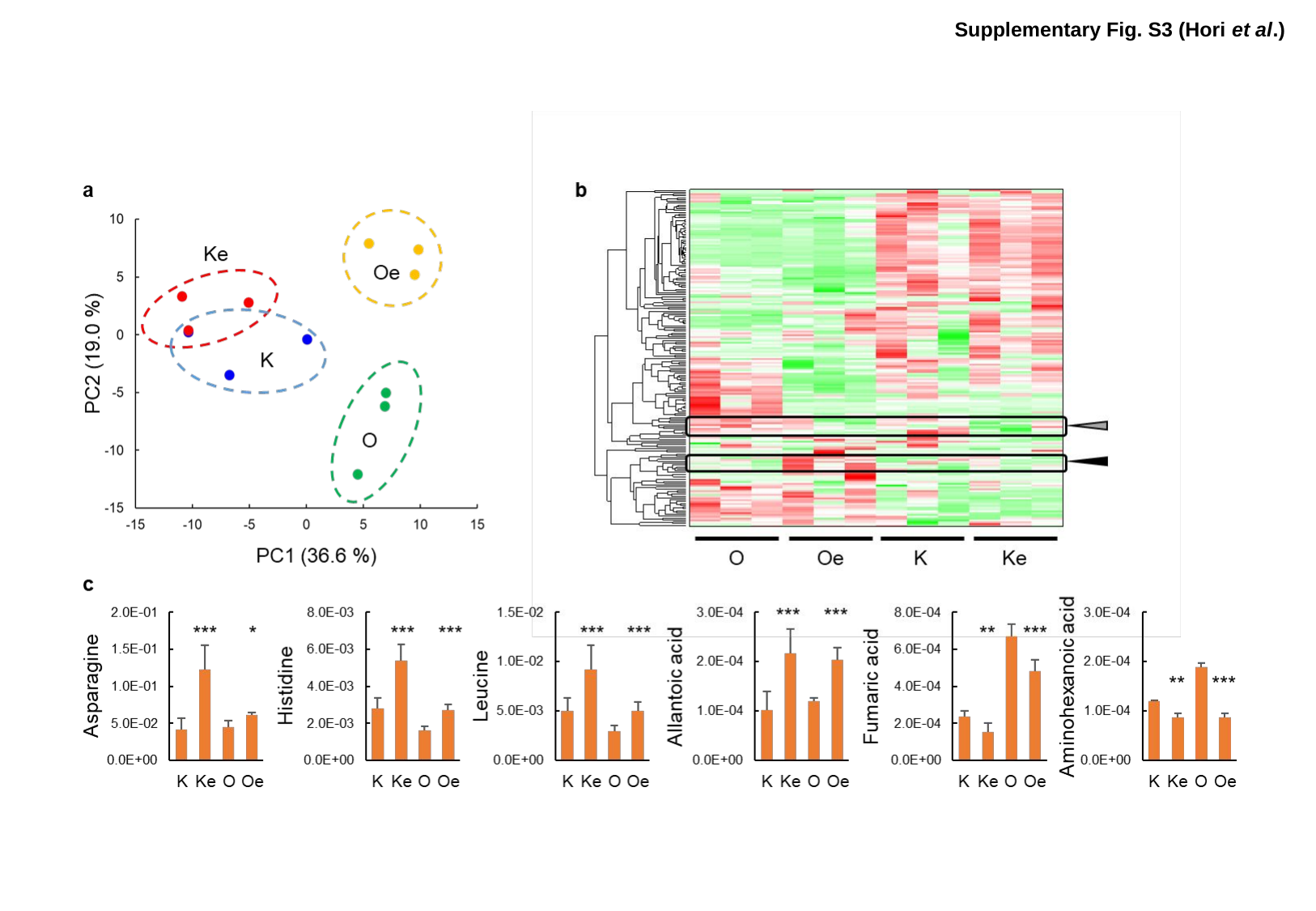

Supplementary Fig. S3 (Hori et al.)

## Slide 4
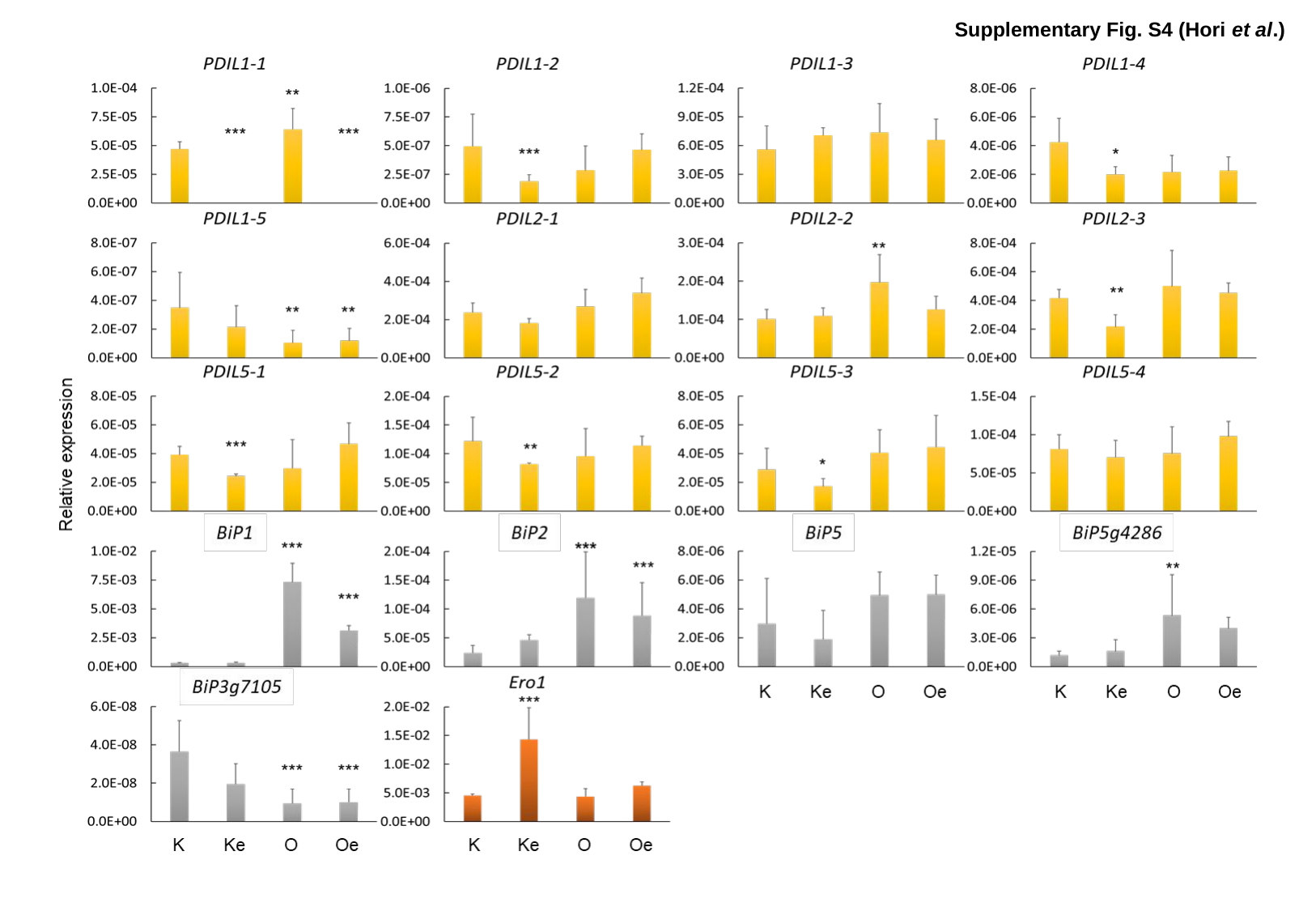

Supplementary Fig. S4 (Hori et al.)

## Slide 5
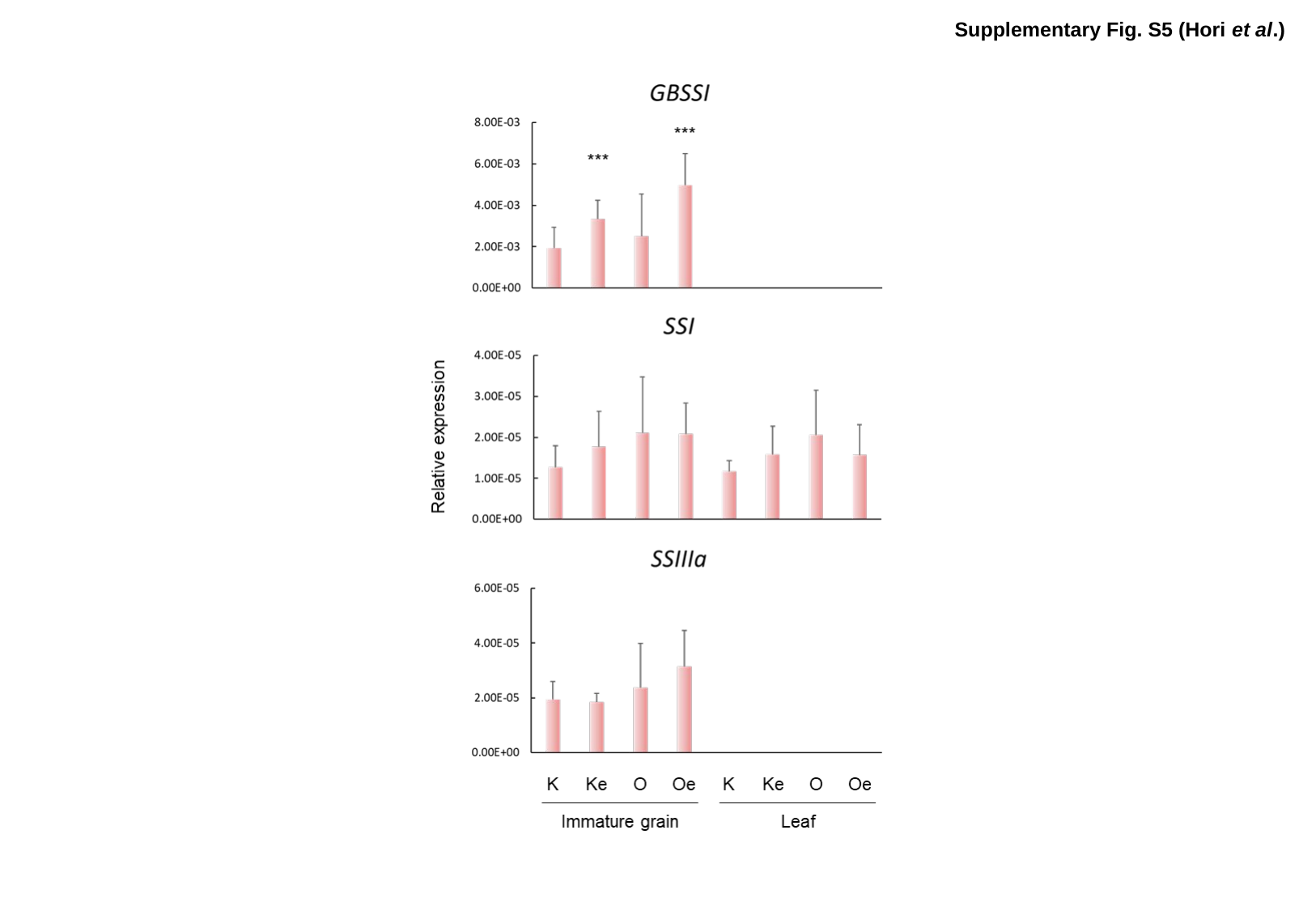

Supplementary Fig. S5 (Hori et al.)
